# Regulation of the Photon Spectrum on Growth and Nutritional Attributes of Baby-Leaf Lettuce at Harvest and during Postharvest Storage

**DOI:** 10.3390/plants10030549

**Published:** 2021-03-14

**Authors:** Viktorija Vaštakaitė-Kairienė, Nathan Kelly, Erik S. Runkle

**Affiliations:** 1 Department of Horticulture, Michigan State University, 1066 Bogue Street, East Lansing, MI 48824-1325, USA; kellyna5@msu.edu (N.K.); runkleer@msu.edu (E.S.R.); 2 Lithuanian Research Centre for Agriculture and Forestry, Institute of Horticulture, Kaunas Street 30, LT-54333 Babtai, Lithuania

**Keywords:** anthocyanins, DPPH, light-emitting diodes, phenols, white light

## Abstract

The photon flux density (PFD) and spectrum regulate the growth, quality attributes, and postharvest physiology of leafy vegetables grown indoors. However, limited information is available on how a photon spectrum enriched with a broad range of different wavebands regulates these factors. To determine this, we grew baby-leaf lettuce ‘Rouxai’ under a PFD of 200 µmol m^−2^ s^−1^ provided by warm-white (WW; control) light-emitting diodes (LEDs) supplemented with either 30 µmol m^−2^ s^−1^ of ultraviolet-A (+UV30) or 50 µmol m^−2^ s^−1^ of blue (+B50), green (+G50), red (+R50), or WW (+WW50) light. We then quantified growth attributes and accumulated secondary metabolites at harvest and during storage in darkness at 5 °C. Additional +G50 light increased shoot fresh and dry weight by 53% and 59% compared to the control. Relative chlorophyll concentration increased under +UV30, +G50, and especially +B50. At harvest, +B50 increased total phenolic content (TPC) by 25% and anthocyanin content (TAC) by 2.0-fold. Additionally, +G50 increased antiradical activity (DPPH) by 29%. After each day of storage, TPC decreased by 2.9 to 7.1% and DPPH by 3.0 to 6.2%, while TAC degradation was less pronounced. Principal component analysis indicated a distinct effect of +G50 on the lettuce at harvest. However, concentrations of metabolites before and during storage were usually greatest under the +B50 and +R50 treatments.

## 1. Introduction

Controlled-environment agriculture (CEA) enables growers to control environmental and cultural factors, thus extending the growing season and achieving a more uniform crop. CEA encompasses semi- or fully-closed growing structures such as greenhouses or indoor farms (i.e., plant factories) [1]. The typical features of indoor farms are the efficient use of water and fertilizer, automatic air temperature and humidity control, and electric lighting, which enable year-round crop production on a demand basis [2]. The photon flux density (PFD) and spectrum are two of the main environmental factors that influence plant productivity and quality when crops are produced indoors. Solid-state light-emitting diodes (LEDs) have become increasingly popular because of their efficient conversion of electrical energy to light energy for indoor plant cultivation [3]. Moreover, LEDs have many advantages over fluorescent or high-intensity discharge lights such as a longer lifetime, smaller size, and faster switching. Finally, narrow-band LEDs are highly adjustable, allowing growers to regulate photosynthesis, plant morphology, and metabolism at any growth stage [4]. The need to deliver an efficient PFD and spectrum that balances crop development, growth, and quality attributes, while considering energy consumption, necessitates further research on photomorphogenesis and LED technology [5].

Lettuce (*Lactuca sativa*) is one of the most widely produced and consumed leafy greens in CEA because it is easy to prepare, is affordable, and has numerous nutritional benefits [6,7]. Although lettuce has high water content and is low in calories, it enriches the human diet with dietary fiber and important mineral nutrients, such as K, Fe, and Na; vitamins B_9_ (folate), C, and E; polyunsaturated fatty acids; and bioactive compounds such as carotenoids and polyphenols that have antioxidant properties [7]. The consumption of nutrient-rich vegetable products can significantly counter human health problems like obesity and chronic disorders, including diabetes or cardiovascular disease [8,9].

Previously published studies revealed that physiological responses of lettuce are strongly influenced by environmental conditions. Lettuce is a common model plant for photophysiological studies in CEA because of its economic importance, short production cycle, and sensitivity to the photon spectrum. For example, supplementing cool-white fluorescent lamps with blue (B; 400–500 nm, peak = 476 nm) and ultraviolet-A (UV-A; 315-400 nm, peak = 373 nm) LEDs increased anthocyanin content by 11% and 31%, B LEDs increased carotenoid concentration by 12%, and red (R; 600–700 nm, peak = 658 nm) LEDs increased phenolic content by 6% in ‘Red Cross’ baby-leaf lettuce [10]. Additionally, supplemental far-red (FR; 700–800 nm; peak = 734 nm) LEDs increased growth and morphological indices from 14 to 28%; however, the phytochemical contents in plants decreased from 11 to 40%.

More recently, studies have shown that increasing the percentage of B (peak = 449 nm) light decreased the fresh and dry mass of red-leaf lettuce, for example in ‘Rouxai’ by up to 63% and 54%, respectively [11]. The same study reported that green (G; 500–600 nm, peak = 526 nm) light had negligible effects on morphology, foliage coloration, essential nutrients, or sensory attributes, regardless of the B PFD. However, increasing the B PFD increased red foliage coloration and the concentrations of several macronutrients (e.g., nitrogen and magnesium) and micronutrients (e.g., zinc and copper). In another lettuce study, the addition of G (peak = 510 nm), yellow (Y; peak = 595 nm), or orange (O; peak = 622 nm) light to R (peaks = 627 and 660 nm), B (peak = 455 nm), and FR (peak = 735 nm) light did not have a pronounced effect on red-leaf lettuce ‘Red Cos’ or green-leaf lettuce ‘Lobjoits Green Cos’ growth and biomass production [12]. However, the leaf area of green-leaf lettuce increased by 30%, and fresh weight (FW) increased by 22%, under supplemental UV-A (peak = 380 nm) light. Additionally, supplemental UV-A increased the nitrite content of green-leaf lettuce (by 6.4 times). In red-leaf lettuce, UV-A slightly reduced the nitrite content, but increased the nitrate content in leaves by 62% compared to light provided by R + B + FR LEDs, while supplemental G and O light decreased nitrate content by 47% and 58% respectively. Supplemental UV-A, G, Y, and O light had inconsistent and often contrasting effects on additional nutrients, including free amino acids, sucrose, fructose, maltose, oxalic acid, citric acid, and malic acid [12].

Baby-leaf greens have become increasingly popular for consumers as a ready-to-eat salad of immature leaves with attractive colors and shapes, and appealing tastes. Cultivation conditions and plant age at harvest are factors that affect the quality attributes of leafy greens [13]. Plants harvested at the early development stage with meager storage reserves and high metabolic rate (e.g., baby-leaf greens) might deteriorate more rapidly, while more mature plants with high storage reserves and low metabolic rate might have a longer shelf life [14]. Few studies have been published on the effects of pre-harvest lighting on biochemical compounds in greens during postharvest storage. Pre-harvest R light (peak = 660 nm, 80 μmol m^−2^ s^−1^) for 24 h before harvest notably delayed the degradation of aliphatic, indole, and total glucosinolates in Chinese kale *(Brassica oleracea* var. *alboglabra* ‘Bailey’) sprouts during postharvest storage, compared to those grown under white (W) light. Additionally, vitamin C was remarkably higher in plants treated with R light on the first and second day after harvest [15]. In another study, pre-harvest lighting with R + W light or monochromatic B light decreased the nitrate content in lettuce ‘Lollo Bionda’ [16]. In the same study, the nitrate content in wild rocket (*Diplotaxis tenuifolia*) decreased following pre-harvest lighting from R + W, R + B, and R + W + FR light.

The effect of pre-harvest LED lighting on the nutritional quality of leafy greens at harvest, and especially during postharvest storage, remains unclear. Further investigations are needed to determine whether a photon spectrum for leafy greens production indoors can maximize their nutritive value at harvest and during storage. The bewildering array of choices, complications, and interactions caused by using different narrow-bandwidth LEDs for indoor crop production encourages research on plant responses to W LEDs, which, depending on amounts and proportions of particular phosphors, emit B, G, R, and, to a small extent, FR light [5]. We grew baby-leaf ‘Rouxai’ lettuce under warm-white (WW) LEDs or with supplemental UV-A, B, G, R, or WW light to determine the effects of supplemental lighting on growth, morphological indices, accumulation of secondary metabolites, and antioxidant activity at harvest and during short-term storage in darkness at 5 °C. We postulated that supplemental lighting treatments of UV-A or B would increase the nutritional traits at harvest and after postharvest storage, while additional G, R, or FR would increase growth but have little, no, or negative effects on nutritional content.

## 2. Results

### 2.1. Shoot Weight and Plant Morphology

**Figure 1:**
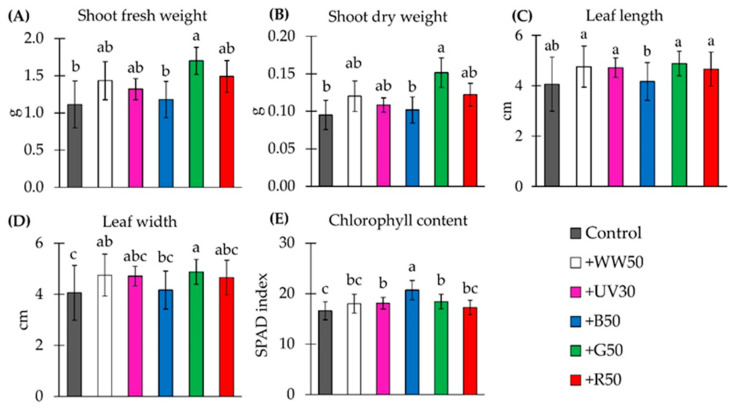
Growth and morphological parameters and chlorophyll content of ‘Rouxai’ baby-leaf lettuce on harvest day. Plants were grown under six lighting treatments at a photon flux density of 200 µmol m^−2^ s^−1^ from warm–white (WW; peak = 639 nm) LEDs (control treatment) without or with an additional 30 µmol m^−2^ s^−1^ of UV-A (peak = 385 nm) (+UV30) or 50 µmol m^−2^ s^−1^ of WW, blue (B; peak = 449 nm), green (G; peak = 526 nm) or red (R; peak = 664 nm) (+WW50, +B50, +G50, and +R50, respectively) light. For shoot fresh weight (**A**), shoot dry weight (**B**), leaf length (**C**), leaf width (**D**), and chlorophyll content (**E**), data are means ± standard deviation (SD) of two replications with 10 samples per replication (*n* = 20). The third leaf was measured for leaf length and width (**C**,**D**). Means with different letters are significantly different from control treatment at the α = 0.05 level by Tukey’s honestly significant difference test.

On harvest day, shoot FW of baby-leaf ‘Rouxai’ lettuce was the highest under the +G50 treatment [200 µmol m^−2^ s^−1^ of WW supplemented with 50 µmol m^−2^ s^−1^ of green (G) light] (Figure 1A). The +G50 treatment significantly increased shoot FW by 53% compared to the control (WW). Compared with the control, there was no significant effect on shoot FW when plants were grown under the +UV30, +WW50, +B50, and +R50 treatments (200 µmol m^−2^ s^−1^ of WW supplemented with 30 µmol m^−2^ s^−1^ of UV-A, or with 50 µmol m^−2^ s^−1^ of WW, blue (B), and red (R) light, respectively). Similarly, plants grown under the +G50 treatment had increased shoot dry weight (DW) by 59%, and there were no significant differences between other lighting treatments on shoot DW compared to the control (Figure 1B). Finally, the leaf length of baby-leaf lettuce under the control was similar to those under the +WW50, +UV30, +B50, +G50, and +R50 lighting treatments (Figure 1C). However, the +WW50 and +G50 treatments expanded leaf width by 17% and 20%, respectively, compared to the control (Figure 1D).

### 2.2. Chlorophyll Concentration

Non-destructive SPAD index measurements showed an increase in relative chlorophyll content in baby-leaf lettuce grown under +UV30 (by 9%), +B50 (by 25%), and +G50 treatments (by 11%) compared to the control (Figure 1E). The SPAD index of plants grown under +WW50 and +R50 treatments was similar to plants grown under the control treatment.

### 2.3. Total Phenolic Content

**Figure 2:**
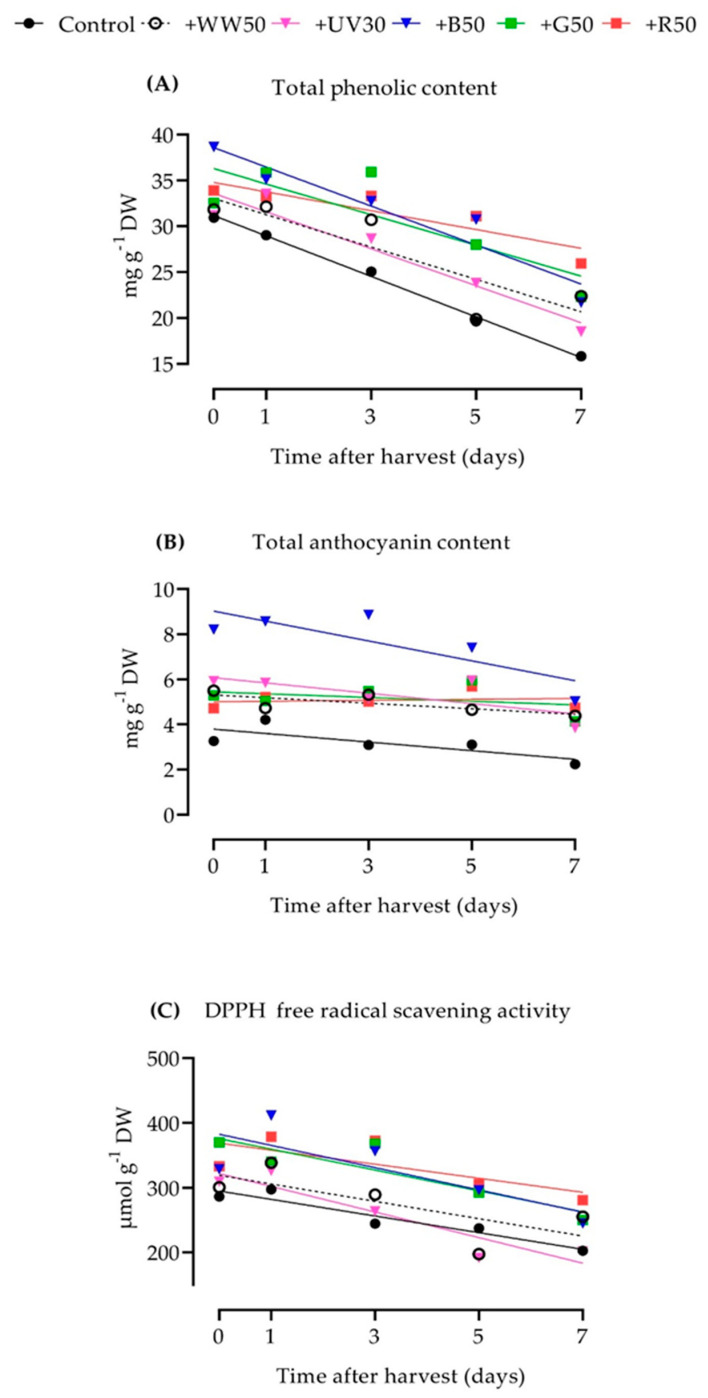
Concentrations of total phenolic content (**A**), total anthocyanin content (**B**), and DPPH free radical scavenging activity (**C**) in ‘Rouxai’ baby-leaf lettuce from harvest until 7 d after harvest. See Table 2 for a description of treatments. Data are presented as the mean of two replications with three randomly selected plants and three analytical measurements per sample (*n* = 6). Linear regression was performed for each treatment and compound.

There were no significant differences in total phenolic content (TPC) in baby-leaf lettuce under the lighting treatments at harvest, except for plants grown under the +B50 treatment, where TPC significantly increased by 25% compared to the control (Table S2). The application of any supplemental lighting treatment decreased the rate at which TPC decreased during postharvest storage. For instance, TPC of control plants decreased by 7.1% per day of postharvest, but only by 5.5% or 5.4% when 50 µmol m^−2^ s^−1^ of WW (+WW50) or B (+B50) light was added to the control (Figure 2A, Table S2). Finally, +R50 led to the greatest preservation of TPC, with a rate of decrease of only 2.9% per day, resulting in the highest TPC at the end of storage.

### 2.4. Total Anthocyanin Content

Similar to TPC, the greatest total anthocyanin content (TAC) in ‘Rouxai’ baby-leaf lettuce on harvest day was in plants grown under the +B50 treatment, which was about 2.0-fold higher than plants grown under the control treatment (Table S1). Furthermore, the +B50 treatment led to 26 to 38% higher TAC than in the other supplemental lighting treatments. However, while +B50 was the most effective at increasing TAC at harvest, it was not necessarily the most effective at preserving TAC during postharvest storage (Figure 2B, Table S2); it decreased by 4.9% per day, which was similar to or less than the 5.7% decrease per day that occurred when plants were grown under the control treatment but more than other supplemental lighting treatments. TAC in plants grown under +WW50, +UV30, and +G50, decreased at an average daily rate of 2.3%, 3.8%, and 1.5%, respectively, and did not decrease when grown under the +R50 treatment. Finally, +R50 and +B50 provided to the plants during the growing period led to some of the highest TACs by the end of storage, while for the control, it led to the lowest concentrations.

### 2.5. DPPH Free Radical Scavenging Activity

On harvest day, lettuce grown under +G50 had 29% greater 2.2-Diphenyl-1-picrylhydrazyl free radical scavenging activity (DPPH) compared to the control (Table S1). However, supplemental UV-A (+UV30), or additional WW, B, and R (+W50, +B50, +R50, respectively) light did not significantly increase DPPH activity compared to the control treatment. Although supplemental G light was the most effective at increasing DPPH free radical scavenging activity at harvest, lettuce plants grown under +R50 had among the lowest rates of decrease in DPPH activity during 7 d of postharvest storage (Figure 2C, Table S2). Those plants decreased in DPPH activity by 3.0% per day of storage, while control plants and those grown under the +B50, +G50, and +WW50 decreased at a daily rate of 4.1% to 4.5%. Leaves from plants grown under the +UV30 treatment had the sharpest decline in DPPH activity at 6.2% per day of storage. Finally, the +R50 treatment led to the greatest preservation of DPPH activity by the end of storage, while +UV30 led to the lowest.

### 2.6. Principal Component Analysis

**Figure 3:**
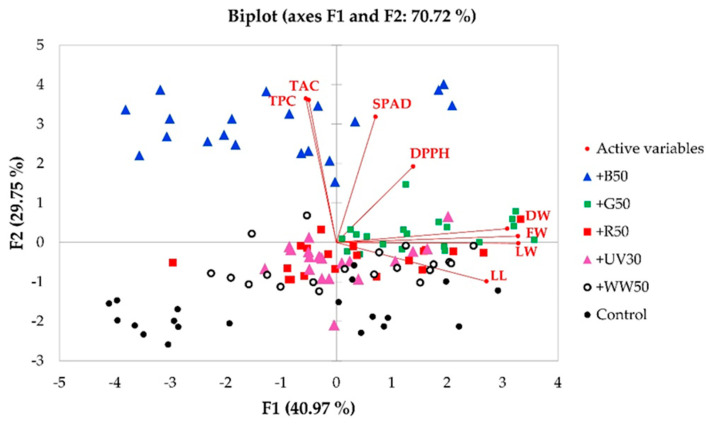
A principal component analysis biplot, indicating distinct effects of lighting treatments on ‘Rouxai’ baby-leaf lettuce and correlations between growth, morphological, and biochemical measurements. LL—leaf length; LW—leaf width; FW—fresh weight; DW—dry weight; DPPH—2.2–Diphenyl–1–picrylhydrazyl free radical scavenging activity; TPC—total phenolic content; TAC—total anthocyanin content. See Table S2 for the description of treatments.

The principal component analysis (PCA) biplot (Figure 3) presents the effects of the six lighting treatments on plant growth, morphological and biochemical parameters, as well as the relationships between FW, DW, leaf length, leaf width, relative chlorophyll content, DPPH, TPC, and TAC in ‘Rouxai’ baby-leaf lettuce on harvest day. The screen plots (Figure S1) of the PCA showed that the first two eigenvalues accounted for most of the variance in the dataset. The PCA factor loadings, scores, and eigenvalues for the first two principal components (F1 and F2) are presented in Table 1. In a PCA biplot, two vectors with an angle < 90° show a positive correlation, and two vectors with an angle > 90° have a negative correlation. The first two PCAs extracted from the components amounted to 70.72% of the total data variance.

To evaluate the associations between biometric measurements and lighting treatments, the PCA biplot was analyzed according to F1 and F2 factor loadings and scores. The PCA indicates that shoot FW and DW were most closely associated with the +G50 treatment. That treatment was also associated with SPAD index and antiradical activity based on DPPH measurements. Leaf length and width of baby-leaf lettuce were associated with the +UV30 and +R50 treatments, as well as +WW50. The TPC and TAC were associated with +B50. In general, the PCA biplot showed a distinct effect of +G50 from the control on growth, morphological, and biochemical parameters of baby leaf lettuce. Additionally, lighting treatments of +WW50, +UV30, and +R50 showed distinct effects in comparison to the control along with F1, and the treatments +B50 and +G50 differed from the control treatment along with F2. The vectors of the PCA biplot showed a significant positive correlation between shoot FW and DW (*r =* 0.821), shoot FW and leaf width (*r =* 0.839), and shoot DW and leaf width (*r =* 0.710) (Table S3). For biochemical compounds, there was a strong correlation between SPAD index and TAC (*r =* 0.601) and between TAC and TPC (*r =* 0.699). DPPH measurements in lettuce did not strongly correlate with any of the biochemical parameters.

## 3. Discussion

Plants have developed sophisticated ways to detect, adapt, and survive in a changing light environment. Plants interpret the detailed information from different wavelengths through the action of unique photoreceptors that are categorized into five main classes. Phytochromes primarily perceive R/FR light (600–750 nm); cryptochromes, phototropins, and F-box containing flavin binding proteins (e.g., ZEITLUPE, FKF1/LKP2) primarily respond to B and UV-A light (315–500 nm); and UVR8 is a UV-B (280–315 nm) photoreceptor [17,18]. These photoreceptors are directly involved in light perception and modulation of morphogenesis and plant biomass. They also regulate the accumulation of specialized metabolites, many of which have applications in the food or pharmaceutical industries [19].

Our experimental results demonstrate generally similar growth and leaf morphological responses of ‘Rouxai’ baby-leaf lettuce to each supplemental waveband of LED lighting. However, the addition of G light increased FW and DW, as well as leaf width compared to the control treatment. Although R and B light drive CO_2_ fixation primarily in the upper leaf layers, G light can better penetrate leaves and increase CO_2_ fixation in the lower leaf cells, leading to greater biomass accumulation [20,21,22,23]. Our results are in agreement with several other studies; for example, R + B LEDs supplemented with 24% of G light from fluorescent lamps (delivering a total PFD of ≈150 µmol m^−2^ s^−1^) increased shoot FW and DW of ‘Waldmann’s Green’ lettuce compared to only R + B LEDs and cool-white or G fluorescent light at the same PFD [24]. In another study, the effects of G light on growth and morphological indices of red-leaf lettuce ‘Banchu Red Fire’ depended on how G light was delivered [25]. At a PFD of 300 µmol m^−2^ s^−1^, the growth of lettuce ‘Banchu Red Fire’ was greater when delivered by G LEDs with a peak = 510 nm than fluorescent fixtures. In red romaine lettuce ‘Outredgeous’, early growth was more vigorous under W with supplemental G light, leading to significantly higher FW than the W-light control 14 d after sowing [26]. In contrast, biomass accumulation of ’Green Oak Leaf’ lettuce shoots was similar among W and W + G lighting treatments at a comparatively low PPF of 135 µmol m^−2^ s^−1^. [27]. A more recent study indicated that the inclusion of G light did not affect shoot DW at a B PFD of 0 or 20 µmol m^−2^ s^−1^, but decreased it when the B PFD was 60 or 100 µmol m^−2^ s^−1^ [11]. However, the partial substitution of R light in a B + R light background (at PFDs of 20 and 160 µmol m^−2^ s^−1^, respectively) with 60 µmol m^−2^ s^−1^ of G light did not influence shoot DW of lettuce ‘Rouxai’ grown for 11 d. Conversely, at day 25, substituting 60 µmol m^−2^ s^−1^ of G light for R decreased shoot FW and DW by 19% [28]. In another study, there were no significant growth differences in ‘Red Cos’ baby-leaf lettuce when grown under R + B + FR LEDs and 30 µmol m^−2^ s^−1^ of R light was substituted by 30 µmol m^−2^ s^−1^ of G light (peak = 510 nm) to [12]. These conflicting reports indicate inconsistent effects of G light on lettuce growth, which could be because of response differences among lettuce varieties, or other factors such as effects of other wavebands (e.g., B light), different total PFDs, plant densities, and plant maturities at measurement.

Chlorophylls are green pigments in plants that are located in chloroplasts and drive photosynthesis by absorbing light and converting it into chemical energy [29]. Recently, the general phenomenon of chlorophyll hormesis among plant species and stress-inducing agents was confirmed. Hormesis is a phenomenon by which a stressor stimulates a cellular stress response, including metabolite production to help organisms establish adaptive responses. It has been suggested that chlorophyll biosynthesis in response to stress is biphasic, with the capacity to equip plants with stress-coping mechanisms by increasing chlorophyll concentrations above homeostatic levels [30]. Chlorophyll stimulation by low-dose stress occurs in tandem with enhanced growth and productivity as well as enhanced photosynthesis. Our results indicated that +UV30, +G50, and +B50 treatments increased relative chlorophyll content (SPAD index) in ‘Rouxai’ baby-leaf lettuce. Although UV-A light can damage the photosynthetic apparatus [31,32], recent studies of maximum quantum yield of photosystem II (PSII) photochemistry of dark- and light-adapted plants, and non-photochemical fluorescence quenching, showed photo-inhibition under supplemental UV-A (peak = 367 nm) to B (peak = 447 nm) and R (peak = 665 nm) LEDs, whereas the photosynthetic response under B + R LEDs with or without additional UV-A (peaks = 387 nm and 402 nm) light did not damage PSII [33]. In agreement, ‘Klee’ lettuce grown under additional UV-A (peak = 365 nm) at a PFD of 10, 20, and 30 µmol m^−2^ s^−1^ had similar leaf photosynthetic rates as those grown under B + R + FR light at a PFD of 237 µmol m^−2^ s^−1^ [34]. In another study, chlorophyll content in ‘Yanzhi’ and ‘Red Butter’ lettuce treated with 10 µmol m^−2^ s^−1^ of supplemental UV-A (peak = 380 nm) to R (peak = 660 ± 10 nm) and W (peak = 440 nm) LEDs at a ratio of 2:3 was remarkably higher than under R + W, R + W + FR, or R + W + UV-A + FR LEDs [35]. Similarly, supplemental UV-A light (peaks = 366 nm, 390 nm, and 402 nm) increased the chlorophyll index of red pak choi (*Brassica rapa* var. *chinensis*) ‘Rubi F_1_’ at a PFD of 6 µmol m^−2^ s^−1^ [36].

Blue light generally increases the formation of chlorophyll (Chl) a, resulting in a relatively high Chl a:b ratio [37]. B light increases gene expression of magnesium chelatase (MGCH) H subunit, glutamyl-tRNA reductase (GluTR), and ferrochelatase (FECH), which promotes chlorophyll synthesis [38,39]. B light also induces chlorophyll biosynthesis via the cryptochrome family of photoreceptors. Cryptochromes 1 and 2 have been both shown to affect gene expression of HEMA1 (glutamyl-tRNA reductase 1) and CHLH (magnesium-chelatase subunit) in the chlorophyll biosynthesis pathway in *Arabidopsis thaliana* [40]. In addition to our results, several studies with leafy green vegetables showed the promotive effect of B light on increased chlorophyll content. In ‘Green Oak Leaf’ lettuce, Chl a and Chl b content were both about 49% higher in plants grown under W + B light compared to those grown under W light alone (total PFD 135 μmol m^−2 ^s^−1^) [27]. In ‘Kinshun’ green cabbage (*Brassica olearacea* var. *capitata*) seedlings, 50 µmol m^−2^ s^−1^ of monochromatic B light (peak = 470 nm) provided for 30 d increased the chlorophyll content [41]. In Chinese cabbage (*Brassica campestris*), B LEDs (peak = 460 nm) alone and in combination with R LEDs (peak = 660 nm) at a total PFD of 80 µmol m^−2^ s^−1^ also increased chlorophyll content [42].

The metabolic profile of fresh vegetables describes their nutritional quality and indicates their potential impact on human health [43]. Phenolic compounds such as flavonoids, including flavonols and anthocyanins, have greater antioxidant activity than vitamin C and tocopherols [44]. Phenolic compounds also play an important role as a defense mechanism against plant stresses. For example, flavonoids are the strongest singlet oxygen and hydrogen peroxide scavengers and are therefore capable of preventing photoinhibition caused by photosynthetic or UV light [45]. Phenolic acids are the main phenols in green vegetables, while anthocyanins are more common in darker-colored vegetables such as red-leaf lettuce varieties [46]. In the present study, we measured significantly higher TPC and TAC in ‘Rouxai’ baby-leaf lettuce cultivated under +B50 compared to the control treatment. B light-induced phenolic synthesis in leafy greens has been previously reported. For example, B light (peak = 468 nm) with or without R light (peak = 655 nm) at a total PFD of 100 µmol m^−2^ s^−1^ increased polyphenol content as well as total antioxidant capacity in ‘Banchu Red Fire’ lettuce seedlings [47]. In addition, TAC in ‘Outredgeous’ red-leaf lettuce was significantly higher in the presence of B light (peak = 440 nm). Furthermore, TPC was over twice as high in plants grown in the presence of B light at a PFD of 30 µmol m^−2^ s^−1^ than in plants grown under R (peak = 640 nm) LEDs alone (total PFD 300 µmol m^−2^ s^−1^) [48]. Finally, B (peak = 476 nm) LEDs (PFD 130 µmol m^−2^ s^−1^) supplemented to fluorescent lamps (total PFD ≈300 µmol m^−2^ s^−1^) increased TAC in ‘Red Cross’ lettuce compared to those grown under only fluorescent lighting [10].

Antioxidant activity is related to concentrations of secondary metabolites that have antioxidant potential, such as phenolic compounds, including anthocyanins. Although our results demonstrate that B light was the most effective at increasing concentrations of compounds that have antioxidant properties, the highest antiradical activity, according to the DPPH free radical scavenging activity assay, was measured in lettuce grown under the +G50 treatment. This result is consistent with a study with ‘De Lier’ butterhead lettuce, in which G light (peak = 530 nm) combined with R (peak = 660 nm) and B (peak = 460 nm) light at a total PFD of 200 µmol m^−2^ s^−1^ increased free radical scavenging activity [49]. Similarly, in wheat (*Triticum aestivum*) and lentil (*Lens esculentum*), the addition of G light (peak = 510 nm) at a PFD of 15 µmol m^−2^ s^−1^ to B (peak = 455 nm), R (peaks = 638 and 669 nm), and FR (peak = 731 nm) light at a total PFD of 200 µmol m^−2^ s^−1^ increased DPPH free radical scavenging activity [50].

Factors that affect the postharvest quality of leafy greens are very broad. In general, postharvest techniques aim to prevent visual, textural, and nutritional deterioration of food that occurs rapidly after harvest [51]. Our results suggest that even though supplemental B light increased contents of phenols, including anthocyanins, and DPPH free radical scavenging activity, the most at harvest, R light was among the most effective at preserving secondary metabolites during post-harvest storage. Few studies have determined the effects of the photon spectrum on the nutritional quality of leafy vegetables during postharvest storage; many more have been conducted at harvest [51]. In agreement with our results, R light suppressed the degradation of TPC and phenolic compounds in Chinese kale sprouts (*Brassica oleracea* var. *alboglabra* ‘Bailey’) during postharvest storage [15]. Consequently, the antioxidant activity of sprouts was elevated by the R light treatment. The differences in phytochemicals of lettuce grown under various lighting treatments during the postharvest storage appear to be a phenomenon that is likely linked not only to the metabolite content at harvest but also to the content of other components, e. g., nutrients or water. However, more studies are needed to clarify the mechanisms to explain the changes in metabolite contents of lettuce that were not consistent among different lighting treatments.

## 4. Materials and Methods

### 4.1. Chemicals

Potassium chloride (KCl; ACS, ≥ 99.0%), sodium acetate (CH_3_COONa; ACS, ≥99.0%), hydrochloric acid (HCl; ACS, ≥37.0%), methanol (≥99.9%), sulfuric acid (H_2_SO_4_; ACS, ≥99.5–98.0%), Folin & Ciocalteu’s phenol reagent (F–C reagent), sodium carbonate (Na_2_CO_3_; ≥99.0%), gallic acid (GA; anhydrous), sodium bicarbonate (NaHCO_3_; ACS, ≥99.7%) were purchased from Sigma–Aldrich (Merck KGaA, Darmstadt, Germany). 2.2–Diphenyl–1–picrylhydrazyl (DPPH reagent; 95%, Alfa Aesar, Haverhill, MA, USA) was purchased from Thermo Fisher Scientific Inc. (Waltham, MA, USA).

### 4.2. Plant Material and Growth Conditions

Experiments were performed in the Controlled–Environment Lighting Laboratory at Michigan State University, East Lansing, MI, USA. On 27 July 2019 (Rep. 1) and 10 September 2019 (Rep. 2), seeds of red oakleaf lettuce ‘Rouxai’ (*Lactuca sativa*) (Johnny’s Selected Seeds, Winslow, ME, USA) were sown into 200–cell (2.5 cm × 2.5 cm) Rockwool cubes (AO 25/40 Starter Plugs; Grodan, Milton, ON, Canada), pre-soaked in deionized water with an adjusted pH of 4.4–4.5 using diluted (1:31) 95–98% sulfuric acid (J.Y. Baker, Inc., Phillipsburg, NJ, USA). Seeded cubes were placed in plastic trays and covered with transparent humidity domes, which were removed 4 d after seed sow. The air temperature was controlled at a constant 22 °C by a ventilation and air-conditioning unit (HBH030A3C20CRS; Heat Controller, LLC., Jackson, MI, USA) that was controlled by a wireless thermostat controller (Honeywell International, Inc., Morris Plains, NJ, USA) A total PFD of 180 µmol m^−2^ s^−1^ from WW (peak = 639 nm, correlated color temperature = 2700 K) LEDs (PHYTOFY RL, OSRAM, Beverley, MA, USA) was delivered for 18 h d^−1^. Seedlings were watered with a water-soluble fertilizer (Jack’s Nutrients FeEd 12–4–16 (N–P–K); JR Peters, Inc. Allentown, PA, USA) and magnesium sulfate (Pennington Epsom salt, Madison, GA, USA) to supply the following nutrients (in mg L^−1^): 125 N (117 NO_3_; 8 NH_4_), 42 P, 167 K, 73 Ca, 47 Mg, 34 S, 0.21 B, 0.21 Cu, 1.6 Fe, 0.5 Mn, 0.01 Mo, and 0.36 Zn. The pH and the electrical conductivity (EC) of the nutrient solution were measured daily using a portable meter (GroLine HI9814, Hanna Instruments, Woonsocket, RI, USA), and was adjusted using H_2_SO_4_ or NaHCO_3_ to maintain a pH of 5.6, and the EC of 1.2 mS cm^−1^.

### 4.3. Production Culture and Environment

For each treatment, 30 lettuce seedlings were transplanted into 36-cell polystyrene rafts (61 × 122 × 2.5 cm; Beaver Plastics; Acheson, AB, Canada) and placed on floating beds (Active Aqua; New Hudson, MI, USA) in a deep-flow hydroponic system with three vertically stacked layers (Indoor Harvest, Houston, TX, USA) on 9 August 2019 (Rep. 1) and 21 September 2019 (Rep. 2). Plants were grown at an air temperature of 22 °C (controlled as described previously) with ambient CO_2_ and under an 18 h d^−1^ photoperiod (0400–2200 HR). Each hydroponic system contained a nutrient solution composed of deionized water and the same fertilizer as described previously but elevated by 20% (e.g., 150 mg L^−1^ N), which was recirculated and oxygenated with an air stone (20.3 × 2.5 cm; Active Aqua AS8RD; Hydrofarm, Petaluma, CA, USA) and a 60–W air pump (Active Aqua AAPA70L; Hydrofarm). The pH (5.6 ± 0.4 standard deviations) and EC (1.7 ± 0.3 mS cm^−1^) of the nutrient solution tanks were measured daily as described previously and adjusted using H_2_SO_4_ or NaHCO_3_. Thermocouples (0.13–mm type E; Omega Engineering, Inc., Stamford, CT, USA), infrared temperature sensors (OS36–01–K–80 F; Omega Engineering, Inc.), light quantum sensors (LI-190R; LI-COR, Inc., Lincoln, NE, USA), CO_2_ sensors (GMD20; Vaisala, Inc., Louisville, CO, USA), and relative humidity and temperature probes (HMP110; Vaisala, Inc.) were used to monitor corresponding environmental parameters. One or two sensors of each type were positioned in representative locations of the growth room.

### 4.4. Lighting Treatments

After transplant, plants were grown under six different lighting treatments delivered by UV-A (peak = 385 nm), blue (B; peak = 449 nm), green (G; peak = 526 nm), red (R; peak = 664 nm), and WW (peak = 639 nm) LEDs, each of which was independently controlled and housed in the same fixture (PHYTOFY RL, OSRAM, Beverley, MA, USA) (Table 2). The PFD of each color channel was controlled at a 1 μmol m^−2^ s^−1^ increment using proprietary software (Spartan Control Software; OSRAM, Beverley, MA, USA). Three LED fixtures (67.3 × 29.8 × 4.3 cm each) were positioned 47 cm above each raft and spaced on 41-cm centers to achieve reasonably good PFD uniformity. Lighting treatments consisted of 200 µmol m^−2^ s^−1^ of WW light (control) without or with an additional 30 µmol m^−2^ s^−1^ of UV-A (+UV30) or 50 µmol m^−2^ s^−1^ of WW, B, G, or R light (+WW50, +B50, +G50, or +R50, respectively) for 18 h d^−1^ (Table 2). The photon distributions of all lighting treatments were measured using a portable spectroradiometer (PS200; Apogee Instruments, Inc., Logan, UT, USA).

### 4.5. Postharvest Storage Conditions

For the postharvest portion of this experiment, five fresh-cut baby-leaf lettuce plants from each lighting treatment were harvested 17 d after seed sow and stored in clear 23 × 24 × 8 cm clamshell polyethylene terephthalate (PET) containers in darkness at 5 °C and 70% relative humidity for 1, 3, 5, and 7 d.

### 4.6. Biometric Measurements and Water Content Determination

After 17 d from seed sow, destructive measurements were conducted on ten baby-leaf lettuce plants from each lighting treatment and replication. Each plant was cut from the Rockwool cube and shoot FW (g) and DW (g) were measured using an analytical balance (AG245; Mettler Toledo, Columbus, OH, USA). Leaf length (cm) and width (cm) of the third fully expanded leaf, and leaf number (when >2 cm) were measured. Shoots were dried in an oven (Blue M, Blue Island, IL, USA) at 60 °C for 4 d before DW measurements. Water content was calculated as the fraction of the difference between shoot FW and DW in FW and used for the re-calculation of biochemical compound contents in the DW of plants.

### 4.7. Non-Destructive Estimation of Chlorophyll Content

The relative chlorophyll content (SPAD) was evaluated using an MC–100 m (Apogee Instruments, Inc, Logan, UT, USA) based on ratio measurements of light transmittance from red and near-infrared wavelengths. Three measurements were made on the third fully expanded leaf of ten plants from each lighting treatment, from two replicates of the experiment, to calculate an average SPAD value. The results are presented as means of SPAD indexes.

### 4.8. Biochemical Analysis

For each lighting treatment within a replication, conjugated biological samples of the third leaf from three randomly selected plants were used for biochemical analysis. Three analytical replicates were performed for each biochemical measurement. Fresh plant tissue was immediately frozen with 10 mL liquid nitrogen (N_2_) and stored in an ultra-low freezer at −80 °C until analysis.

#### 4.8.1. Quantification of Total Phenolic Content

The total phenolic content (TPC) of lettuce was determined spectrophotometrically [52] with slight modifications. Frozen fresh plant tissue (500 mg) was homogenized in a ceramic mortar with 5 mL of 80% ice-cold methanol and transferred to a 15 mL polypropylene conical centrifuge tube (Falcon, Thermo Fisher Scientific Inc.). The extract was incubated at 4 °C for 24 h. Samples were centrifuged (Heraeus Megafuge, Thermo Fisher Scientific Inc.) at a relative centrifugal force of 4000× *g* for 5 min at room temperature, and the supernatant was filtered through a 70 mm qualitative filter paper (Whatman Qualitative Filter Paper Grade 1, Thermo Fisher Scientific Inc.). 100 µL of the filtrate was diluted with 200 µL of 10 % (vol/vol) F–C reagent and vortexed thoroughly. Then, 800 µL of 700 mM of Na_2_CO_3_ was added. The prepared analytical samples were covered and left to stand for 20 min. The absorbance of the samples was measured using a spectrophotometer (BioSpec–mini; Shimadzu, Japan) at 765 nm. The TPC in fresh plant tissues was calculated using a standard curve of GA (*R*^2^ > 0.95). Data are presented as the mean of three analytical measurements of TPC (in mg g^−1^) on a dry basis of the plant.

#### 4.8.2. Quantification of Total Monomeric Anthocyanin Pigment Content 

Total anthocyanin content (TAC) was determined by the pH differential method (AOAC Official Method 2005.2) [53] with slight modifications. We used the molar extinction coefficient of 34,300 L cm^−1^ M^−1^ by using molecular weight 449.2 g M^−1^ for cyanidin-3-glucoside for measurements of TAC in frozen fresh plant powder (300 mg) prepared with 1% HCl in methanol [54]. The method is based on the anthocyanin structural transformation that occurs with a change in pH (colored at pH 1.0 and colorless at pH 4.5). The prepared samples were incubated at 4 °C for 48 h. Then, samples were centrifuged as previously described, and the supernatant was filtered through filter paper as previously described. Two separate analytical samples were prepared to measure TAC in the plant extract. 400 µL of the extract was mixed with 2 mL of 0.025 M KCl and 2 mL of 0.4 M CH₃COONa, and after 20 min, the absorbance was measured at 530 nm and 700 nm using the same spectrophotometer. The dilution factor was 6. Data are presented as a mean of three analytical TAC measurements (in mg g^−1^) on a dry basis of the plant.

#### 4.8.3. Evaluation of Antiradical Activity by 2.2–Diphenyl–1–Picrylhydrazyl Free Radical (DPPH) Scavenging Activity Method

The antiradical activity was evaluated according to the spectrophotometric DPPH scavenging activity method [55,56] with modifications. 100 µL of 80% methanol extracts used for the TPC assay were diluted with 1 mL of 60 µM DPPH solution. The absorbance was measured after 16 min using the same spectrophotometer at 515 nm. The ability of plant extract to scavenge DPPH free radicals was calculated using the DPPH solution as a blank. Data are presented as the mean of three analytical samples to scavenge DPPH free radicals (in µmol g^−1^) on a dry basis of the plant.

### 4.9. Statistical Analysis

The experiment was arranged as a randomized complete block design with two replications (blocks) in time. Statistical analysis was conducted using R statistical analysis software (version 3.5.1; R Foundation for Statistical Computing, Vienna, Austria). Analysis of variance (ANOVA) and Tukey’s honestly significant difference test (α = 0.05) was performed using the R packages ‘dplyr’ [57] and ‘agricolae’ [58] and compared with the control treatment. For the ANOVA of biochemical compounds of plants under storage conditions, new samples were taken each day of investigation (1, 3, 5, and 7 d after the harvest), and compared to samples under control (WW) on the same day. Finally, linear regression analysis was performed on the biochemical compound concentrations during storage using SigmaPlot 14.0 (Systat Software, Inc, San Jose, CA, USA).

### 4.10. Principal Component Analysis

Principal component analysis (PCA) was performed using Microsoft^®^ Excel^®^ for ‘Microsoft 365’ and Addinsoft XLSTAT 2020 statistical and data analysis solution (Long Island, NY, USA). The PCA was performed at a 95% significance level. The results presented in the PCA biplot indicate distinct effects of lighting treatments on plant growth and morphology parameters or contents of biochemical compounds, and Pearson’s correlation matrix summarized relationships between biometric measurements or investigated biochemical compounds in baby leaf lettuces under the lighting treatments.

## 5. Conclusions

The addition of light from UV-A, B, G, R, or WW LEDs differently regulated ‘Rouxai’ baby-leaf growth and leaf morphology. However, the addition of G light led to the greatest plant growth and leaf width as well as chlorophyll content in lettuce leaves. In addition, plants grown under UV-A, and especially B light, had significantly higher relative chlorophyll content. Supplemental UV-A and B light promoted the accumulation of secondary metabolites in baby-leaf lettuce, but significant changes in TPC and TAC were in plants grown under supplemental B light. However, the highest antioxidant activity (according to DPPH free radical scavenging activity) was in lettuce grown under supplemental G light. During postharvest storage, the TPC and DPPH free radical activity decreased, and the TAC degradation was lower. Despite that, supplemental B light increased the contents of secondary metabolites at harvest, while R light was among the most effective at preserving phytochemicals during short-term postharvest storage.

## Figures and Tables

**Table 1:** Factor loadings, eigenvalues, variability (%), cumulative variability (%), and scores for the first two principal (F1–F2) components for growth, morphological, and biochemical measurements of ‘Rouxai’ baby-leaf lettuce grown under six different lighting treatments on harvest day. Lighting treatments consisted of a photon flux density of 200 µmol m^−2^ s^−1^ from warm-white (WW) LEDs (control) without or with an additional 30 µmol m^−2^ s^−1^ of UV-A (+UV30) or 50 µmol m^−2^ s^−1^ of WW, blue (B), green (G), or red (R) light (+WW50, +B50, +G50, or +R50, respectively) for 18 h d^−1^.

Factors	F1	F2
Eigenvalue	3.278	2.380
Variability (%)	40.97	29.75
Cumulative variability (%)	70.72	
Factor Loadings		
FW	0.923	0.039
DW	0.868	0.084
LL	0.763	−0.237
LW	0.924	−0.005
SPAD	0.199	0.764
DPPH	0.390	0.463
TAC	−0.140	0.867
TPC	−0.155	0.875
Factor Scores		
Control	−1.106	−1.773
+WW50	0.237	−0.595
+UV30	0.029	−0.461
+B50	−1.110	2.974
+G50	1.559	0.229
+R50	0.392	−0.375

FW—shoot fresh weight; DW—shoot dry weight; LL—leaf length; LW—leaf width; SPAD—relative chlorophyll concentration (index); DPPH—2.2–Diphenyl–1–picrylhydrazyl free radical scavenging activity; TAC—total anthocyanin content; TPC – total phenolic content.

**Table 2:** LED types and photon flux densities (PFD) were used to deliver six lighting treatments used in experiments.

Lighting Treatments	PFD, µmol m^−2^ s^−1^						DTPFDI
	Warm-White (WW)	UV-A (UV)	Blue (B)	Green (G)	Red (R)	Total	
**Control**	200					200	12.9
+WW50	250					250	16.2
+UV30	200	30				230	14.9
+B50	200		50			250	16.2
+G50	200			50		250	16.2
+R50	200				50	250	16.2

DTPFI—daily total photon flux density integral, mol m^−2^ d^−1^.

## Supplementary Materials

The following are available online at https://www.mdpi.com/2223-7747/10/3/549/s1, Table S1: Total phenolic content, total anthocyanin content, and antiradical activity of ‘Rouxai’ baby-leaf lettuce plants under different lighting treatments on harvest day and during postharvest storage, Table S2: Coefficients for linear regression equations presented in Figure 3, in the form of y = y0 + ax, and their correlation coefficients (R^2^), Table S3: Correlation matrix (Pearson) between biometric and biochemical measurements of baby leaf lettuce, Figure S1: Scree plot of biometric and biochemical measurements of baby leaf lettuce under different lighting treatments.
