# Supplementary material for: Regulation of the Photon Spectrum on Growth and Nutritional Attributes of Baby-Leaf Lettuce at Harvest and during Postharvest Storage
Source: Plants (Basel). 2021 Mar 14;10(3):549. doi: 10.3390/plants10030549 (PMC8000317; doi:10.3390/plants10030549)

Supplementary material

Table S1. Total phenolic content, total anthocyanin content, and antiradical activity of 'Rouxai' baby-leaf lettuce plants grown under different lighting treatments on harvest day and during postharvest (PH) storage in darkness at 5 °C.

Radiation treatments	TPC, mg g⁻¹ DW	TAC, mg g⁻¹ DW	DPPH, µmol g⁻¹ DW
Harvest day			
WW	30.95 [b]	4.21 [c]	286.40 [b]
+WW50	31.82 [b]	5.50 [b]	300.57 [b]
+UV30	31.20 [b]	5.92 [b]	309.22 [ab]
+B50	38.65 [a]	8.21 [a]	328.37 [ab]
+G50	32.53 [b]	5.29 [bc]	369.76 [a]
+R50	33.91 [b]	4.72 [bc]	332.95 [ab]
1–day PH			
WW	29.02 [b]	3.27 [c]	297.35 [c]
+WW50	32.15 [ab]	4.73 [bc]	338.58 [bc]
+UV30	33.53 [ab]	5.84 [b]	326.15 [bc]
+B50	35.10 [a]	8.57 [a]	411.52 [a]
+G50	35.86 [a]	5.04 [b]	340.05 [bc]
+R50	33.23 [ab]	5.22 [b]	378.49 [ab]
3–days PH			
WW	25.06 [c]	3.09 [b]	244.45 [b]
+WW50	30.72 [b]	5.32 [b]	289.32 [b]
+UV30	28.66 [bc]	5.14 [b]	263.56 [b]
+B50	32.74 [ab]	8.87 [a]	356.31 [a]
+G50	35.93 [a]	5.48 [b]	367.89 [a]
+R50	33.30 [ab]	5.01 [b]	372.33 [a]
5–days PH			
WW	19.63 [b]	3.10 [c]	237.45 [ab]
+WW50	19.87 [b]	4.65 [bc]	197.47 [ab]
+UV30	23.81 [ab]	5.95 [ab]	191.76 [b]
+B50	30.72 [a]	7.41 [a]	296.13 [ab]
+G50	28.01 [ab]	5.95 [ab]	292.29 [ab]
+R50	31.11 [a]	5.68 [ab]	305.76 [a]
7–days PH			
WW	15.82 [b]	2.24 [b]	202.73 [a]
+WW50	22.38 [ab]	4.39 [a]	255.15 [a]
+UV30	18.52 [ab]	3.85 [ab]	201.78 [a]
+B50	21.69 [ab]	5.03 [a]	244.83 [a]
+G50	22.33 [ab]	4.14 [a]	250.05 [a]
+R50	25.94 [a]	4.74 [a]	280.68 [a]

TPC – total phenolic content; TAC – total anthocyanin content; DPPH – 2.2–Diphenyl–1–picrylhydrazyl free radical scavenging activity; 1–day PH to 7–days PH – number of days after the harvest. Plants were grown under six lighting treatments of 200 µmol m^{-2} s^{-1} from warm–white (WW; peak = 639 nm) LEDs (treatment WW; control) without or with an additional 30 µmol m^{-2} s^{-1} of UV–A (peak = 385 nm) or 50 µmol m^{-2} s^{-1} of WW, blue (B; peak = 449 nm), green (G; peak = 526 nm) or red (R; peak = 664 nm) light from LEDs (+WW50, +B50, +G50, and +R50, respectively). Data are presented as the mean of two replications with three randomly selected plants and three analytical measurements per

sample. Means with different letters are significantly different from the control treatment at the $\alpha < 0.05$ level by Tukey's honestly significant difference test.

Table S2. Coefficients for linear regression equations presented in Figure 2, in the form of y = y0 + ax, and their correlation coefficients (R^2).

Lighting treatments	TPC			TAC			DPPH		
	y0	*a*	R^2	*y0*	*a*	R^2	*y0*	*a*	R^2
Control	31.2	-2.2	0.996	3.88	-0.22	0.802	295	-13	0.916
+WW50	33.0	-1.8	0.757	5.31	-0.12	0.555	319	-13	0.521
+UV30	33.6	-2.0	0.919	6.07	-0.23	0.530	322	-20	0.862
+B50	38.6	-2.1	0.915	9.03	-0.44	0.665	382	-17	0.617
+G50	36.3	-1.7	0.684	5.44	-0.08	0.121	376	-16	0.792
+R50	34.8	-1.0	0.804	5.01	0.02	0.020	369	-11	0.544

TPC – total phenolic content; TAC – total anthocyanin content; DPPH – 2.2–Diphenyl–1–picrylhydrazyl free radical scavenging activity. Lighting treatments consisted of a photon flux density of 200 μmol m^{-2} s^{-1} from warm-white (WW) LEDs (control) without or with an additional 30 μmol m^{-2} s^{-1} of UV–A (+UV30) or 50 μmol m^{-2} s^{-1} of WW, blue (B), green (G), or red (R) light (+WW50, +B50, +G50, or +R50, respectively) for 18 h d^{-1}.

Table S3. Correlation matrix (Pearson) between biometric and biochemical measurements of baby leaf lettuce.

Variables	FW	DW	LL	LW	SPAD	DPPH	TAC	TPC
FW	**1**	**0.821**	**0.562**	**0.839**	0.145	**0.337**	-0.076	-0.090
DW	**0.821**	**1**	**0.460**	**0.710**	0.176	**0.403**	-0.069	-0.074
LL	**0.562**	**0.460**	**1**	**0.741**	0.083	0.087	**-0.265**	**-0.294**
LW	**0.839**	**0.710**	**0.741**	**1**	**0.194**	**0.210**	-0.064	-0.130
SPAD	0.145	0.176	0.083	**0.194**	**1**	**0.221**	**0.601**	**0.493**
DPPH·	**0.337**	**0.403**	0.087	**0.210**	**0.221**	**1**	0.154	**0.356**
TAC	-0.076	-0.069	**-0.265**	-0.064	**0.601**	0.154	**1**	**0.699**
TPC	-0.090	-0.074	**-0.294**	-0.130	**0.493**	**0.356**	**0.699**	**1**

Values in bold are different from 0 with a significance level $\alpha = 0.05$. FW – shoot fresh weight; DW – shoot dry weight; LL – leaf length; LW – leaf width; SPAD – relative chlorophyll content (index); DPPH – 2.2–Diphenyl–1–picrylhydrazyl free radical scavenging activity; TAC – total anthocyanin content; TPC – total phenolic content.

Figure S1. Scree plot of biometric and biochemical measurements of baby-leaf lettuce grown under different lighting treatments.

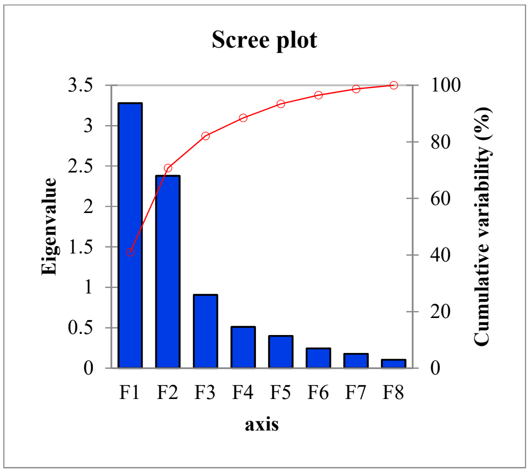

Supplement: Supplementary file 1 [file plants-10-00549-s001.pdf]
